# Postoperative Outcomes of Laparoscopic Hepatic Resection Combined With Radiofrequency Ablation for Two or Three Hepatocellular Carcinomas: A Case Series of Nine Patients

**DOI:** 10.7759/cureus.73864

**Published:** 2024-11-17

**Authors:** Masahiro Yamamoto, Kazue Morishima, Kazuhiro Endo, Kentaro Shimodaira, Yuki Kimura, Yuichi Aoki, Masanobu Taguchi, Hideki Sasanuma, Yasunaru Sakuma, Shunji Watanabe, Naoki Morimoto, Naohiro Sata

**Affiliations:** 1 Department of Surgery, Division of Gastroenterological, General and Transplant Surgery, Jichi Medical University, Shimotsuke, JPN; 2 Department of Internal Medicine, Division of Gastroenterology and Hepatology, Jichi Medical University, Shimotsuke, JPN

**Keywords:** hepatocellular carcinoma (hcc), laparoscopic hepatic resection, laparoscopic liver resection, laparoscopic radiofrequency ablation, radiofrequency ablation (rfa)

## Abstract

Introduction: Hepatic resection (HR) and radiofrequency ablation (RFA) are curative treatments for three or fewer hepatocellular carcinomas in Japan. The laparoscopic approach in both has been used in recent years; however, its treatment outcome in combination with HR with RFA is unclear. We aimed to gain insights into this combined treatment.

Case presentation: This was a retrospective study of nine patients with two to three hepatocellular carcinomas who had undergone laparoscopic HR combined with laparoscopic RFA between December 2014 and February 2022. Six patients tested positive for hepatitis C virus, two had alcoholic cirrhosis, and one had non-alcoholic steatohepatitis. All patients had a Child-Pugh score of 5 (A). All 22 lesions were treated as planned. Laparoscopic HR was performed on 12 and laparoscopic RFA was performed on 10 lesions. Postoperative hemorrhage occurred in one patient and was managed conservatively. The remaining eight patients were discharged without complications. Recurrence occurred in the residual liver in eight patients. However, no local recurrence at the treatment site was noted during the observation period (15-94 months).

Conclusion: The treatment combining laparoscopic HR and RFA was feasible for the local control of two to three HCCs and may be useful for preserving residual liver function.

## Introduction

Liver cancer ranked sixth among global cancer diagnoses (4.7%) and was the third leading cause of cancer-related deaths (8.3%) in 2020 [1]. Hepatocellular carcinoma (HCC) is the most common primary hepatic malignancy. Regional differences exist in the incidence of HCC, which is common in Africa and East Asia. In Japan, in 2019, the incidence rate per 100,000 individuals was 29.6, ranking seventh among malignant neoplasms, and the number of deaths due to HCC was the fifth highest among cancer-related deaths [2].

Most cases of HCC occur in a background of chronic diseases, such as viral or alcoholic hepatitis. While liver transplantation (LT) can be a curative treatment, it is not feasible in some cases because of insufficient donor livers. Therefore, many patients experience tumor recurrence after treatment. A long-term strategy that considers the possibility of post-treatment recurrence is important when selecting therapy options.

Most treatment guidelines for HCC advocate hepatic resection (HR) or radiofrequency ablation (RFA) as local curative treatments for early-stage disease [3-5]. Each method has its advantages and disadvantages. The treatment is selected based on the size, location, and surrounding vasculature of the lesion. Therefore, the ideal treatment strategy may differ for each lesion in patients with multiple lesions. However, these guidelines do not describe the combination of HR and RFA. Nevertheless, several attempts have been made to combine them with an open approach, and the results have been favorable [6-10].

Recent reports have highlighted the advantages of the laparoscopic approach in HR and RFA [11-14]. Therefore, it is clinically important to determine whether a combination of the two can provide effective local control. However, this issue has not been adequately investigated. Demonstrating effective local control with this combined treatment could greatly benefit patients.

In this retrospective study, we aimed to review the data of nine patients with two to three HCCs treated with laparoscopic HR combined with laparoscopic RFA.

## Materials and methods

This retrospective study was approved by the Jichi Medical University Hospital Ethics Committee (approval number: A21-064).

Patients

We retrospectively reviewed nine patients with HCC who underwent laparoscopic HR combined with laparoscopic RFA at Jichi Medical University Hospital from December 2014 to February 2022. All patients were treated based on the clinical practice guidelines for HCC provided by the Japan Society of Hepatology [3].

The patients ranged in age from 60 to 79 and comprised six men and three women. Of these, six tested positive for the hepatitis C virus, two had alcoholic cirrhosis, and one had non-alcoholic steatohepatitis. Three patients had previously received treatment, including non-laparoscopic HR, percutaneous RFA, and transarterial chemoembolization (TACE), for HCC. All patients had a Child-Pugh score of 5 (A). Two of the nine were treated with an anticoagulant for atrial fibrillation and underwent preoperative heparin replacement.

Preoperative imaging assessments were performed using ultrasonography, contrast-enhanced computed tomography, and contrast-enhanced magnetic resonance imaging. These assessments revealed three lesions in four patients and two in five. Among the 22 lesions, 19 were 30 mm or smaller in diameter, while the remaining three measured 35, 42, and 100 mm. The descriptive details are shown in Table 1.

**Table 1:** Baseline characteristics and treatment of all included patients HCV: hepatitis C virus; RFA: radiofrequency ablation; TACE: transarterial chemoembolization

Case #	Age (years)	Sex	Etiology	Child-Pugh	Past therapy	Location and size of the lesions	Anticoagulant therapy
1	67	F	HCV	A	-	S5 20 mm, S7 10 mm	-
2	68	M	HCV	A	-	S8 15 mm, S8 15 mm, S5 7 mm	-
3	77	M	HCV	A	-	S1 20 mm, S5 11 mm, S4 8 mm	+
4	77	F	HCV	A	Percutaneous RFA, TACE	S6 10 mm, S4 15 mm	-
5	66	M	Alcohol	A	-	S6 42 mm, S8 9 mm	-
6	60	M	Alcohol	A	-	S2/3 100 mm, S4/8 10 mm, S7 17 mm	+
7	66	M	HCV	A	S6 resection	S3 15 mm, S5 5 mm	-
8	69	F	HCV	A	Percutaneous RFA	S1 22 mm, S3 13 mm, S2/3 8 mm	-
9	79	M	Non-alcoholic steatohepatitis	A	-	S8 35 mm, S7 12 mm	-

Selection criteria of HR and RFA

Twenty-two lesions were selected for laparoscopic HR or RFA based on the clinical practice guidelines for HCC provided by the Japan Society of Hepatology [3]. The selection criteria for laparoscopic HR were as follows: tumors larger than 30 mm, lesions located near the liver surface or major vascular or biliary structures, and difficulty in securing a puncture route for RFA.

**Figure 1:**
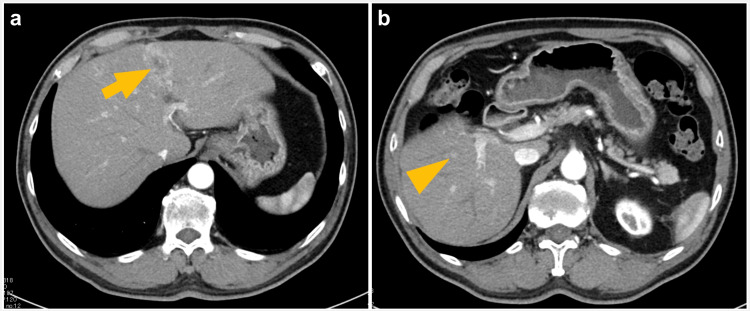
Preoperative assessment identified two lesions in a patient with hepatocellular carcinoma (case 7) (a) The S3 lesion (13 mm), indicated by an arrow, was adjacent to the portal vein and bile duct, leading to the selection of partial resection as the treatment. (b) The S5 lesion (5 mm), indicated by an arrowhead, was deeply located, prompting the selection of radiofrequency ablation.

Laparoscopic RFA was chosen based on the following criteria: tumors smaller than 30 mm, lesions located deep in the liver and away from major vascular or biliary structures, and lesions without vascular invasion. Figure 1 shows examples of the lesions indicated to be treated by laparoscopic HR and laparoscopic RFA, respectively.

Laparoscopic HR and RFA procedures

All procedures were performed under general anesthesia, and a pneumoperitoneum was established using carbon dioxide. Laparoscopic procedures were typically performed using five access ports. Upon abdominal entry, intraoperative ultrasonography was routinely used to identify the lesions. In cases where lesions were difficult to discern, a contrast agent for ultrasonography (Sonazoid®, Daiichi-Sankyo, Tokyo, Japan) was administered to enhance lesion detection.

Laparoscopic HR

The extent of resection was determined using intraoperative ultrasonography. To manage hepatic blood flow, the Pringle maneuver was employed. Parenchymal transection was performed using either an ultrasonic cavitation device (CUSA®, Integra LifeSciences Limited, Tullamore, Ireland) or the clamp-crushing technique with ultrasonic scissors (Harmonic series, ETHICON ENDO-SURGERY, Cincinnati, Ohio, United States). Finally, Glisson capsules and vessels were secured using clips.

Laparoscopic RFA

Ports constructed for laparoscopic HR were also used for laparoscopic RFA, with additional ports inserted as needed. Tumors were examined in detail using laparoscopic ultrasonography, and a safe insertion route to avoid vessel injury was determined. If necessary, a saline solution was infused to create a fluid space to obtain a sufficient echo window. Under ultrasound guidance and laparoscopic vision, the RFA needle was precisely directed into the target lesion using the guide needle. After adequate ablation of the tumor center, needle-tract cauterization was performed to prevent bleeding.

## Results

All 22 lesions were successfully treated as planned. Laparoscopic HR was performed in 12 lesions. Among them, three had tumor diameters of >30 mm, two were located on the liver surface, and two were deep but close to the portal or hepatic vein. Five lesions were difficult to treat with RFA because of adhesions or tumor location. The procedures comprised one lateral segmentectomy, one sub-segmentectomy, and 10 partial resections. Two of the resected lesions had positive surgical margins. Laparoscopic RFA was performed on 10 lesions, all measuring less than 30 mm and situated in deep locations. The mean distance from the liver surface to the planned RFA lesion was 21.5 mm (8-46 mm). An additional port was required in one case to safely perform laparoscopic RFA. Details of the lesions are shown in Table 1 and Table 2.

**Table 2:** Surgical outcomes RFA: radiofrequency ablation

Case #	Resection	RFA	Location and distance from the liver surface to the planned RFA lesion	Operation time (min)	Blood loss (ml)	Postoperative complication	Postoperative hospital stay (day)	Surgical margin
1	S5 partial resection	S7	S7 45 mm	367	150	-	9	-
2	S5 partial resection, S8 partial resection	S8	S8 15 mm	400	370	-	11	-
3	S1 partial resection, S5 partial resection	S4	S4 46 mm	337	200	-	12	+ (S1)
4	S6 partial resection	S4	S4 26 mm	266	55	Bleeding	13	-
5	S6 partial resection	S8	S8 4 mm	325	20	-	7	-
6	Lateral segmentectomy	S4/8, S7	S4/8 25 mm, S7 23 mm	327	5	-	12	-
7	S3 partial resection	S5	S5 7 mm	331	10	-	12	-
8	S1 partial resection, S3 partial resection	S2/3	S2/3 8 mm	236	50	-	9	+ (S3)
9	S8 subsegmental resection	S7	S7 16 mm	519	420	-	12	-

Outcomes

The mean operative time, blood loss, and length of postoperative hospital stay were 345.3 min, 142.2 mL, and 10.8 days, respectively. One patient showed postoperative bleeding equivalent to Clavien-Dindo classification II. The patient was treated with hemostatic agents and platelet transfusions. The remaining eight patients had no postoperative complications. Details of the outcomes are shown in Table 2.

No local recurrence was identified at the RFA treatment site during the observation period (15-94 months). Recurrence was observed in eight of the nine patients during this period; all recurrences were observed within the residual liver, away from the RFA-treated area. The median time to recurrence was 17 months (3-28 months). Treatments for recurrent lesions were as follows: one patient underwent laparoscopic HR, one underwent HR, four underwent RFA, and five underwent TACE. During the observation period, two patients died of liver failure due to recurrence in the residual liver. Details of the recurrence are shown in Table 3.

**Table 3:** Survival of patients HR: hepatic resection; RFA: radiofrequency ablation; TACE: transarterial chemoembolization

Case #	Recurrence (site)	Duration (M)	Treatment for recurrent site	Prognosis
1	-			86 M alive
2	+ (liver)	27	Percutaneous RFA, TACE	61 M alive
3	+ (liver)	21	Percutaneous RFA, TACE	56 M alive
4	+ (liver)	7	Laparoscopic RFA, radiation	48 M alive
5	+ (liver)	3	TACE, chemotherapy, radiation	72 M alive
6	+ (liver)	8	TACE	40 M dead
7	+ (liver)	22	Laparoscopic HR, laparoscopic RFA	94 M alive
8	+ (liver)	28	TACE, chemotherapy	38 M dead
9	+ (liver)	14	Laparoscopic HR	15 M alive

## Discussion

This study investigated the postoperative outcomes of laparoscopic HR combined with RFA at our hospital. In all patients, treatment was performed as planned, and no local recurrence occurred at the treatment site during the observation period (15-94 months). Liver failure was not observed during the perioperative period. 

In selecting a curative treatment for HCC, the radicality of the lesions and the preservation of residual liver function must be considered. In many countries, treatment guidelines tailored for local situations are often used to select treatment options for HCC. The Barcelona Clinic Liver Cancer Classification (BCLC) is frequently employed in Europe and the United States, and the Japan Society of Hepatology Consensus Statement and Recommendations are employed in Japan [3-5].

The BCLC strategy recommends HR and RFA as local treatments for single HCC. LT may be considered if patients have no contraindications to LT. However, HR, RFA, and TACE are options for three or fewer tumors determined based on liver function and tumor size in Japan. LT is not recommended in cases of proper liver function owing to the shortage of available donors. Despite differences in staging, both guidelines recommend HR and RFA as curative treatments for localized lesions. We adhered to the Japan Society of Hepatology Consensus Statement and Recommendations, opting to perform HR and RFA instead of TACE for local control in patients who have a tolerance for HR and RFA.

HR and RFA have their respective advantages and disadvantages. HR can be performed even if the tumor is in contact with the vascular system, and there is no technical limit on the tumor diameter that can be operated upon. However, normal liver tissue is also removed, which affects remnant liver function [15,16]. Particularly when the tumor is in a deep location, the risk of postoperative liver failure is high due to the unavoidable resection of normal liver tissue [17]. In contrast, RFA can treat deep lesions with minimal damage to the residual liver but is limited to tumor size [18]. Thus, HR and RFA may complement each other's disadvantages depending on the location and size of the tumor.

For patients with multiple tumors, an appropriate approach must be selected for each lesion based on its characteristics. Therefore, it is reasonable to combine HR and RFA, depending on the case. Although there are no guideline recommendations for combination therapy, previous studies have examined its efficacy and safety [6-10]. Huang et al. compared the short- and long-term outcomes of patients with multifocal tumors after HR with or without intraoperative RFA [9]. No significant difference was observed in overall survival, recurrence-free survival, and postoperative complications between the two groups. These studies state that combination treatment is feasible and safe and may offer curative opportunities for complicated tumor distribution; however, they are all "open" approaches.

Recently, a laparoscopic approach has been used for HR and RFA. This approach has several advantages over open or percutaneous approaches. Laparoscopic HR is less invasive as compared with open surgery, with small abdominal incisions and less intraoperative bleeding due to pneumoperitoneum [11,12]. Laparoscopic RFA can provide a safe puncture route to liver lesions that are difficult or impossible to treat with percutaneous RFA [13]. Despite these advantages and feasibility, the combined treatment using the laparoscopic approach is not a standardized technique, and only a few studies have been reported in the literature [11]. In our study, all the patients underwent laparoscopic HR and laparoscopic RFA safely; they had no postoperative liver failure or local recurrences at the treatment site during the observation period. Thus, this approach may have a relatively low impact on liver function and contribute to effective local control.

In contrast, eight of the nine patients had recurrence, and all new lesions were observed in the remnant liver. As HCC often develops in the liver due to various chronic diseases, heterochronous HCC often occurs in the remnant liver. To avoid recurrence within a short postoperative period, selecting suitable patients for combination therapy is necessary. Han et al. developed a monogram accurately predicting the recurrence-free survival of patients with HCC who undergo radical treatment using preoperative factors [19]. Early local treatment may be avoided in patients expected to have early recurrence. In addition, preservation of residual liver function is important to preserve treatment options at the time of recurrence because HCC often recurs. As systemic therapies, such as molecular-targeted agents and immunotherapies, are advancing, this approach that preserves residual liver function will become increasingly important.

This study had some limitations. First, this case series was a retrospective study involving a small number of patients at a single institution. Additionally, it included cases with a short observation period, which may underestimate long-term complications. To evaluate the efficiency and safety of laparoscopic HR combined with RFA for HCCs, the conclusions drawn from this study should be verified by further prospective randomized controlled trials with large sample sizes.

## Conclusions

Combining laparoscopic HR and RFA in patients with two or three HCCs is feasible and adequate for local control. This approach may be useful for preserving residual liver function. However, as heterochronous HCC often occurs in the remnant liver, making sure that selected patients are suitable for this therapy is necessary to avoid recurrence within a short postoperative period. To evaluate the efficiency and safety of this therapy, the conclusions drawn from our study should be verified by further prospective randomized controlled trials with large sample sizes.
